# Non-thermal processing of polyphenol- and carotenoid-rich green leafy vegetables for healthy aging: effects on antioxidant properties and bioavailability

**DOI:** 10.3389/fnut.2026.1813542

**Published:** 2026-04-24

**Authors:** Marwa Ezz El-Din Ibrahim, Mihriban Elçiçek, Seydi Yıkmış, Nazan Tokatlı Demirok, Afnan I. Alandanoosi, Huda Aldossari

**Affiliations:** 1 Department of Food and Nutrition Sciences, College of Agricultural and Food Sciences, King Faisal University, Al-Ahsa, Saudi Arabia; 2 Nutrition and Dietetics, Faculty of Health Sciences, Tekirdag Namık Kemal University, Tekirdag, Türkiye; 3 Department of Food Technology, Tekirdag Namık Kemal University, Tekirdag, Türkiye

**Keywords:** bioavailability, green leafy vegetables, healthy aging, HPP, non-thermal processing, PEF

## Abstract

Green Leafy Vegetables (GLVs) are crucial for healthy aging due to their polyphenols and carotenoids; however, thermal processing often compromises the stability and bioavailability of these compounds. Non-thermal technologies offer an alternative to preserve sensitive phytochemicals by modifying cell wall structures to enhance bioaccessibility. This review evaluates the biochemical effects of Pulsed Electric Field (PEF), High Hydrostatic Pressure (HPP), Cold Plasma (CP), and Ultrasound (US) on GLV matrices. Evidence indicates that these methods, unlike thermal treatments, maintain antioxidant capacity and improve interactions with digestive enzymes across various food forms. Although current findings, predominantly from *in vitro* models, demonstrate improved stability and release of bound bioactives, clinical validation in elderly populations is required. Consequently, non-thermal processing represents a significant approach for optimizing the functional quality of GLVs in sustainable nutrition strategies.

## 1 Introduction

The global demographic structure is undergoing a fundamental shift due to rising life expectancy, making the management of age-related chronic diseases and the promotion of healthy aging top priorities. According to data from the World Health Organization (WHO) and the United Nations (UN), global life expectancy has reached 73.3 years, and the population aged 60 and over is projected to reach 1.4 billion by 2030 (1). Epidemiological analyses point to a significant increase in the prevalence of cardiovascular diseases, neurodegenerative disorders, and metabolic syndrome in the aging population, parallel to this demographic shift (2). While healthy aging is defined as the preservation of functional capacity, molecular repair mechanisms weaken with age, and susceptibility to disease increases as immune resistance declines (3). Therefore, it is crucial to understand the biological mechanisms underlying both the natural aging process and age-related pathologies (4).

One of the most distinct indicators of cellular aging is telomere shortening, which accelerates under the influence of oxidative stress and metabolic dysregulation (5). Furthermore, the increase in Reactive Oxygen Species (ROS) resulting from mitochondrial dysfunction is known to activate the NLRP3 inflammasome, causing a chronic, low-grade inflammation termed inflammaging (6). Dietary antioxidants are thought to play a critical protective role against this pathological process by upregulating defense enzymes, particularly through the activation of the Nrf2 signaling pathway (7). Current data indicate that plant-based foods support DNA repair mechanisms by modulating telomerase activity and slowing down the telomere loss associated with aging (8, 9). Plant-based diets centered on vegetables, fruits, and whole grains can yield superior metabolic health outcomes due to the synergistic effect of the whole food rather than isolated components (10, 11). However, the potential of flavonoid- and terpene-rich nutraceuticals, such as *Panax ginseng* and *Ginkgo biloba*, to improve cognitive functions and quality of life is also noteworthy (8, 12). Antioxidants naturally present in plants neutralize cellular damage by stabilizing free radicals, a capacity often analyzed using *in vitro* methods such as 2,2-diphenyl-1-picrylhydrazyl (DPPH), Oxygen Radical Absorbance Capacity (ORAC), 2,2′-azino-bis (3-ethylbenzothiazoline-6-sulfonic acid) (ABTS), and Ferric Reducing Antioxidant Power (FRAP) (13, 14). In particular, GLVs are rich reservoirs of flavonoids, as well as ascorbic acid, tocopherols, and β-carotene (15, 16). The antioxidant support provided by GLV consumption plays a critical role in preventing age-related degenerative diseases by minimizing lipid peroxidation and DNA damage (17).

Although GLVs are rich in bioactive compounds, traditional thermal processing methods such as blanching cause the loss of heat-sensitive vitamins and phenolic compounds. While high-temperature applications reduce microbial load, they lead to thermal degradation of nutrients and the loss of water-soluble components (18). These limitations have increased interest in Non-Thermal Processing techniques that preserve nutritional quality while ensuring food safety (19). Technologies such as CP, HPP, PEF, and US, developed in response to consumer demand for fresh-like products, inactivate oxidative enzymes while maintaining the structural integrity of bioactive components (20, 21). Studies have shown that the US, PEF, HHP, hydrodynamic cavitation (HDC), CP, and dynamic high-pressure microfluidization (DHPM) methods yield better results in preserving bioactives and antioxidant activity than thermal processes (22–25). There are even findings indicating that the controlled stress created by these technologies can enhance secondary metabolite production in plant tissues (26). Based on the role of diet in the aging process and the limitations of traditional processing, this review examines the physical and biochemical changes induced by non-thermal technologies in GLVs. The study discusses the effects of engineering parameters on cell wall modifications and the *in vitro* bioaccessibility of bioactive components using quantitative data. Furthermore, product formulations suitable for the nutritional requirements of elderly individuals are evaluated, and a framework for future research is presented by highlighting the relationship between *in vitro* findings and *in vivo* bioavailability.

## 2 Methods/literature review strategy

This study was prepared to compile and critically synthesize the effects of non-thermal processing technologies (HPP, PEF, CP, US) on the bioactive compound profile, antioxidant potential, and bioaccessibility/bioavailability of polyphenol- and carotenoid-rich GLVs (e.g., spinach, arugula, lettuce, chard, kale, etc.) within the context of healthy aging. The literature review was primarily conducted using the Web of Science Core Collection, Scopus, and PubMed databases. In the search strategy, subject-specific keywords were combined with Boolean operators and scanned in the title, abstract, and keyword fields. The keyword groups used included: (i) target product group: “green leafy vegetables,” “leafy greens,” “spinach,” “lettuce,” “rocket,” “chard,” “kale”; (ii) bioactive components: “polyphenol*,” “phenolic compound*,” “flavonoid*,” “carotenoid*,” “chlorophyll*”; (iii) health outcomes and processes: “healthy aging,” “oxidative stress,” “antioxidant,” “inflammation”; (iv) digestion and bioavailability: “bioaccessibility,” “bioavailability,” “*in vitro* digestion,” “INFOGEST,” “gastrointestinal digestion”; (v) process technologies: “non-thermal processing,” “high pressure processing,” “HPP,” “cold plasma,” “ultrasound,” “thermosonication,” “pulsed electric field,” “PEF.” Search terms were combined, for example, as follows: (“green leafy vegetables” or spinach or lettuce or kale) and (polyphenol* or carotenoid*) and (HPP OR PEF or “cold plasma” or ultrasound or “non-thermal”). Additionally, reference lists of selected key studies were screened (backward chaining), and citation network analysis was performed to reach new publications related to the topic.

Inclusion criteria for the study were defined as follows: (1) conducted on GLVs or products derived from them (fresh/minimally processed, puree/smoothie, fruit-vegetable juice, dried powder, etc.); (2) evaluation of non-thermal technologies or hybrid approaches compatible with non-thermal methods; (3) reporting at least one of the following: total phenolic content, total flavonoids, total carotenoids/chlorophyll, antioxidant capacity (DPPH, ABTS, FRAP, etc.), oxidative stability, enzyme activity [e.g., polyphenol oxidase (PPO), peroxidase (POD)], microbial load, quality parameters (color/texture) and/or bioaccessibility/bioavailability indicators; (4) being a peer-reviewed journal article. Exclusion criteria were defined as: (i) plant groups outside the scope of the studies or not directly related to GLVs, (ii) studies addressing only thermal processing, (iii) publications with inaccessible full texts or insufficient method/result reporting, and (iv) studies limited to abstracts/conference proceedings. Selected studies were classified by technology (HPP, PEF, CP, US); for each technology, process parameters (e.g., pressure/field strength/energy density/time), matrix-specific variables (fresh leaf, liquid product, dried form, etc.), measured outcomes, and the reported direction/magnitude of the effect were evaluated comparatively. Bioaccessibility results (especially *in vitro* digestion models; static/dynamic approaches and the INFOGEST protocol) and bioavailability (*in vivo*/human data) findings were addressed in a separate framework, and the impact of methodological heterogeneity on the generalizability of the results was discussed. Thus, the review aimed not only to summarize existing evidence but also to provide a critical synthesis considering the process–matrix–bioactive–digestion interaction. During the initial search, 297 articles were screened using the defined keywords and databases. The limitations of this methodological search strategy include restricting the search to peer-reviewed articles published in English, even though some may have been published in other languages.

## 3 GLVs: composition and relevance to healthy aging

GLVs generally refer to a group of plants whose leaves, stems, or young shoots are consumed as food and that possess high chlorophyll and water content due to their dense photosynthetic tissues. In terms of botanical diversity, GLVs encompass different families such as *Amaranthaceae, Brassicaceae, Asteraceae,* and *Apiaceae* (Table 1).

**Table 1:** Taxonomic classification and general characteristics of commonly consumed GLVs*.

Family	Examples	Key characteristics	References
*Amaranthaceae*	Spinach (*Spinacia oleracea*), Chard (*Beta vulgaris* subsp. v*ulgaris*), Beet Greens (*Beta vulgaris*)	Rich in betalains and antioxidant pigments; high phenolic and flavonoid content	(101)
*Brassicaceae*	Kale (*Brassica oleracea* var. s*abellica*), Rocket (*Eruca sativa*), Mustard (*Brassica juncea*), Broccoli Greens (*Brassica oleracea var. italica*)	Rich in sulfur compounds (glucosinolates)	(102)
*Asteraceae*	Lettuce (*Lactuca sativa*), Endive/Chicory (*Cichorium endivia* / *Cichorium intybus*), Dandelion (*Taraxacum officinale*)	Contains sesquiterpene lactones and inulin	(103)
*Apiaceae*	Parsley (*Petroselinum crispum*), Coriander (*Coriandrum sativum*), Dill (*Anethum graveolens*)	Rich in essential oils and flavones (apiin)	(104)

These vegetables are considered functional foods due to their rich phytochemical profiles, despite their low energy densities (27). The Food and Agriculture Organization (FAO) and WHO recommend consuming 4–5 portions of vegetables and fruits totaling 400 grams per day to reduce the risk of chronic diseases (28). GLVs are a fundamental component of a balanced diet, characterized by edible leaves that can be consumed raw or processed. Encompassing globally common species such as spinach, lettuce, and cabbage as well as local and traditional plants such as purslane and dandelion, this group holds strategic importance in the human diet due to its high nutrient density and unique sensory properties (29). The polyphenol profile of GLVs is primarily classified into phenolic acids and flavonoids, which are structurally related secondary metabolites commonly found in plant foods. Within this broad phytochemical network, flavonoids, in particular, encompass several bioactive subgroups, including flavonols (e.g., quercetin, kaempferol) and anthocyanins. However, in addition to flavonoids, other polyphenolic structures, such as tannins and lignin, are present in the plant matrix, and these components collectively contribute to the plant’s antioxidant capacity. Cruciferous vegetables (Brassicaceae family) such as cabbage, broccoli, and cauliflower possess a phytochemical matrix rich in glucosinolates and isothiocyanates, which exhibit anticarcinogenic activity, as well as antioxidant micronutrients such as ascorbate, tocopherol, and carotene, which are associated with reduced mortality risk (30). GLVs are quite rich in carotenoid pigments, which give plants their natural color and are vital for human metabolism. Components such as beta-carotene, lycopene, and lutein stand out particularly within this group. As precursors to vitamin A, these pigments play a protective role against cancer, metabolic syndromes, and cumulative cellular damage by neutralizing free radicals thanks to their strong antioxidant properties (31). In addition to carotenoids, these vegetables are also strong sources of vitamin C (ascorbic acid), which supports the immune system, and vitamin E (tocopherol), which protects cell membranes against oxidation. These vitamins interact synergistically with carotenoids, increasing the total antioxidant capacity of the plant matrix (32).

However, it is crucial to recognize that the biological effects of these phytochemicals often follow a biphasic dose–response curve (hormesis). While optimal levels of antioxidants neutralize free radicals and support cellular health, an excessive intake can disrupt the delicate redox balance, potentially leading to pro-oxidant effects or interfering with essential ROS-mediated physiological signaling (33, 34).

Epidemiological data indicate that intake of GLV-derived flavonols (especially kaempferol and quercetin) slows the rate of cognitive decline in older adults, while dietary nitrate content lowers cardiovascular disease risk by supporting vascular health (35, 36). Large-scale prospective studies show that these vegetables are associated with a reduced risk of obesity, diabetes, and overall mortality due to their anti-inflammatory properties. At the clinical level, *in vivo* studies have shown that the high carotenoid content (lutein, beta-carotene) of GLVs delays skin aging by protecting collagen and elastin structures in the dermis (37, 38). The phytochemical profile of GLVs is not static but varies with factors such as genotype, harvest maturity, and agricultural conditions (especially light spectrum and fertilization) (39). In the post-harvest process, mechanical stress, especially from washing, triggers an oxidative response that accelerates vitamin C loss and may increase oxalate levels, an antinutritional component. The integration of Modified Atmosphere Packaging (MAP) and innovative non-thermal processing technologies is critical for maintaining bioactive stability in products with high respiration rates (28, 40). The phytochemical diversity of GLVs and the relationship of these components with aging mechanisms are presented in Table 2.

**Table 2:** Bioactive profiles, physiological effects, and main GLVs sources for healthy aging*.

Bioactive component	Physiological effect	Description	Main GLVs sources
Polyphenols	Enhances antioxidant defense; reduces chronic inflammation, regulates immune response	Supports free radical scavenging and suppresses inflammatory signaling pathways	Spinach, Chard, Kale, Rocket, Lettuce
Carotenoids	Supports vision health; supports cognitive functions, slows cellular aging	Lutein, zeaxanthin, and beta-carotene protect the retina and neural tissue	Spinach, Kale, Chard
Flavonols	Protects cardiovascular health; supports a healthy lifespan	Strengthens antioxidant and metabolic balance	Spinach, Kale, Chard

GLVs, green leafy vegetables.

^*^Based on Asadi et al. (105), Bondonno et al. (35), Čeryová et al. (106), Holland et al. (36), and Jacquier et al. (45).

## 4 Oxidative stress, inflammation, and aging mechanisms

ROS are produced as natural byproducts of cellular respiration and metabolism. However, with aging, increased ROS production, driven by various internal and external factors, overwhelms the organism’s antioxidant defense mechanisms, disrupting redox homeostasis and causing oxidative stress. According to the free radical theory of aging, this persistent state of oxidative stress damages cellular macromolecules (lipids, proteins, DNA), leading to functional losses, and is considered the primary driving force of the aging process (41). Mitochondria, where oxidative phosphorylation generates cellular energy, are at the center of this process. While small amounts of ROS are released as byproducts during Adenosine Triphosphate (ATP) synthesis and are considered normal, oxidative stress resulting from a disrupted balance threatens mitochondrial function (42). Excessive ROS accumulation reduces mitochondrial membrane potential, disrupting energy production, triggering cell death (apoptosis), and impairing organelle structure by suppressing the SIRT1 (Sirtuin 1)/PGC-1α signaling pathway responsible for mitochondrial renewal. Ultimately, this vicious cycle, where damage to the electron transport chain leads to further ROS production, accelerates cellular aging (41). Additionally, with age, impairment of the mitophagy mechanism, which normally clears damaged mitochondria, leads to the accumulation of dysfunctional organelles and deepens the chronic inflammatory cycle. Consequently, the aging process leads to “inflammaging,” characterized by continuous immune system activation and elevated levels of circulating inflammatory markers. This sterile and chronic state of inflammation, arising from the failure of resilience mechanisms against accumulated damage, is accompanied by increases in pro-inflammatory cytokines such as C-Reactive Protein (CRP), Interleukin-6 (IL-6), and Tumor Necrosis Factor-alpha (TNF-α), and is viewed as a primary risk factor for many age-related pathologies (43).

### 4.1 Molecular signaling pathways and their modulation by phytochemicals

The aging process is characterized by dysfunction of critical signaling pathways that regulate cellular homeostasis. Current literature indicates that molecular mechanisms controlling oxidative stress response, chronic inflammation, and energy metabolism play a central role in the development of age-related pathologies (43, 44). The most important line of cellular defense is the Nrf2/Keap1-ARE pathway. Normally, Nrf2 is suppressed by Keap1. However, in the presence of oxidative stress, Nrf2 translocates to the nucleus. There, it initiates the transcription of cytoprotective genes (44).

Bioactives found in GLVs can modulate this critical pathway. For instance, glucoraphanin, present in cruciferous vegetables like kale and broccoli, converts into sulforaphane—a potent Nrf2 activator—via the myrosinase enzyme released upon tissue disruption (45, 46). Due to the high heat sensitivity of myrosinase, non-thermal processing technologies offer a significant advantage over traditional methods in preserving this vital bioconversion (46).

Another critical mechanism is the NF-κB signaling pathway, which controls immunity and inflammation (43). Increased oxidative stress during the aging process causes the NF-κB pathway to remain continuously active, leading to an increase in pro-inflammatory cytokines and the suppression of antioxidant defense (44, 47). Luteolin, a naturally occurring flavonoid prominent in celery and parsley, directly targets neuroinflammation due to its ability to cross the blood–brain barrier. It exhibits a dual effect by activating Nrf2 while suppressing NF-κB, contributing to the management of the inflammaging process (48).

**Figure 1:**
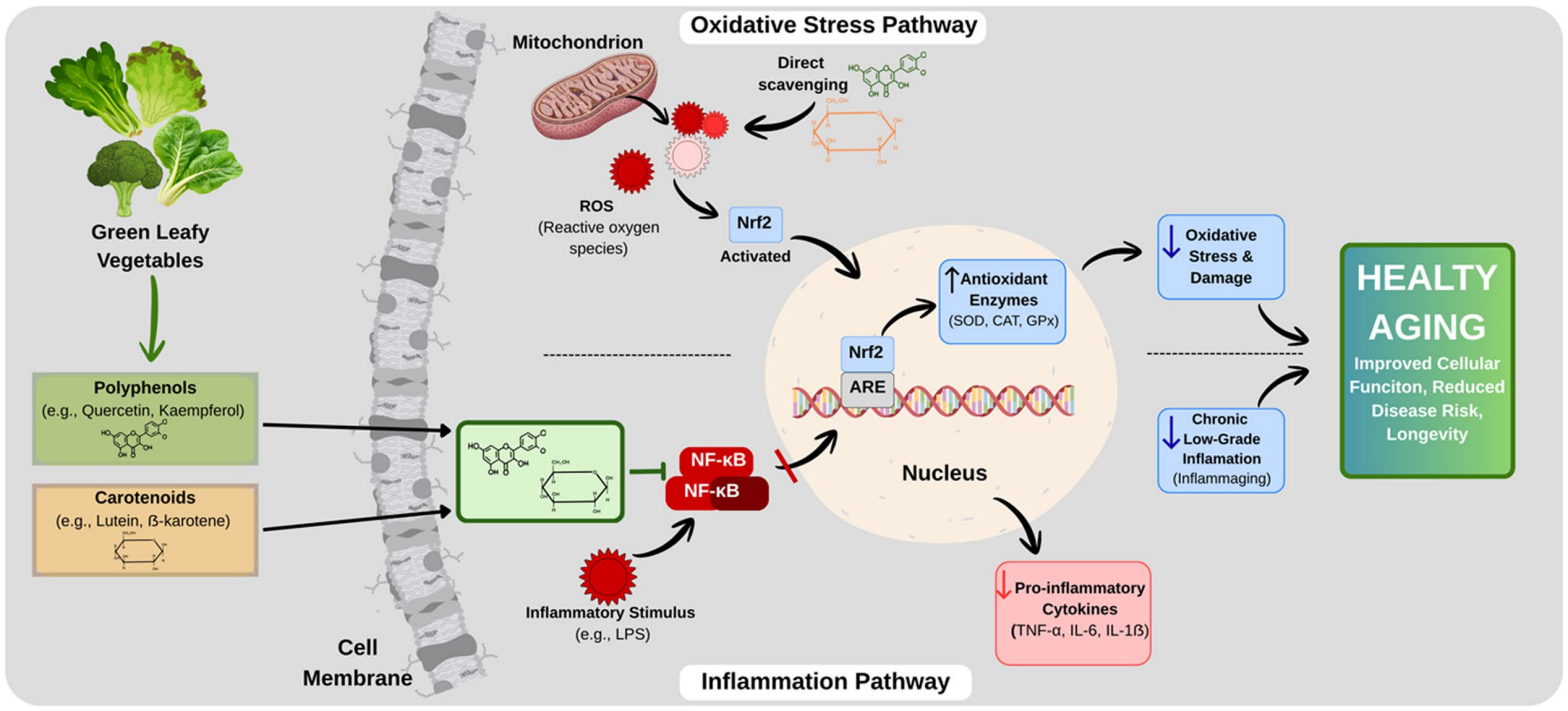
Molecular mechanisms of action of GLVs bioactives in healthy aging. GPx, Glutathione POD; NF-κB, Nuclear factor kappa B; SOD, Superoxide Dismutase; ARE, Antioxidant Response Element; IL-1β, Interleukin-1 beta; IL-6, Interleukin-6; Nrf2, Nuclear factor erythroid 2-related factor 2; LPS, Lipopolysaccharide; CAT, Catalase; TNF-α, Tumor Necrosis Factor alpha.

Other cascades involved in aging biology include the Mitogen-Activated Protein Kinase (MAPK), SIRT1, and the mechanistic Target of Rapamycin (mTOR) pathways. SIRT1 extends lifespan by enhancing mitochondrial biogenesis and suppressing NF-κB, but declining NAD + levels with age weaken this protection (47). As detailed in the ‘Mechanism’ section above, quercetin, which is found in kale, leeks, and other GLVs, supports mitochondrial health by activating SIRT1. It also plays a critical role in tissue regeneration by clearing senescent cells through its senolytic properties (49). On the other hand, the p38 MAPK pathway triggers senescence, while excessive activation of the mTOR pathway accelerates aging. Inhibition of mTOR activates autophagy, ensuring the clearance of damaged components (43, 44). These mechanisms and the regulatory roles of GLVs-derived bioactives are summarized in Figure 1.

## 5 Non-thermal processing technologies

Non-thermal processing technologies, developed to minimize nutrient losses caused by traditional thermal processing, achieve microbial inactivation without raising food temperature to critical levels (typically <40–50 °C) (20). These technologies operate on the principle of hurdle technology to ensure food safety while preserving the fresh-like sensory properties and nutritional value of GLV’s products (50). Among these technologies, US is based on the principle of propagating sound waves above the human hearing threshold (>20 kHz) within the food matrix (51). Pressure fluctuations created in the liquid medium cause the formation and violent collapse of microscopic bubbles (acoustic cavitation) (52). It has been shown that thermosonication, combined with mild thermal processing, is more effective in enzyme inactivation than ultrasound alone and better preserves bioactive components (53). Another important method, HPP, involves applying isostatic pressure ranging from 100 to 600 MPa to packaged food using water (54). This process inactivates enzymes by disrupting the three-dimensional (tertiary) structures of proteins, while it does not damage covalently bound small molecules such as vitamins and pigments. Current reviews confirm that HPP preserves chlorophyll and carotenoid stability by over 90% in sensitive products such as spinach, compared with thermal processing (20, 55). PEF technology applies short-duration, high-voltage (0.5–80 kV/cm) electric pulses to food in the order of microseconds (56). Its fundamental mechanism is to create a critical transmembrane potential on the cell membrane, opening pores (electroporation) (57). Plasma, the fourth state of matter, is a reactive mixture consisting of ionized gasses and UV photons (58).

Short-wavelength Ultraviolet light technology (UV-C, 200–280 nm) inactivates microorganisms by inducing DNA damage, which prevents replication, while stimulating defense mechanisms in plants, potentially increasing polyphenol accumulation (59, 60). The effect of these technologies on the GLV’s matrix primarily occurs through cell wall modification and the breaking of mass transfer resistance. In particular, PEF and US applications create a critical transmembrane potential on the cell membrane, opening temporary or permanent pores (57). This physical change disrupts the selectivity of the semi-permeable membrane, lowers intracellular turgor pressure, and reduces cell wall resistance. Micro-cracks formed in the cellulosic network by the cavitation effect of ultrasound facilitate the entry of digestive enzymes into the cell, initiating the bioavailability process (53). With the loosening of the cell wall and the opening of pores, barriers preventing the outward diffusion of intracellular fluid are removed; this accelerates the transfer of dissolved bioactive metabolites out of the matrix, increasing extraction efficiency (51).

One of the most critical challenges in the processing stage is preserving tissue integrity and preventing enzymatic browning (61). When leaf tissue is damaged, PPO and POD enzymes oxidize phenolics, leading to undesirable color changes (62). However, since non-thermal methods do not detach the magnesium atom at the center of chlorophyll, they provide a major advantage in maintaining the vivid green color of vegetables (55). Beyond all these quality parameters, the environmental footprint of these technologies is gaining importance within the concept of Green Processing (63, 64). Comprehensive analyses show that non-thermal methods (especially PEF) can reduce electricity consumption by 20% and water usage by 25% compared to traditional thermal pasteurization (56). These sustainable features strongly position non-thermal technologies as the future standard in food production (64).

## 6 Effect of non-thermal processing on polyphenols and antioxidant capacity

The most pronounced effect of non-thermal processing on the GLV matrix is a measurable increase in total phenolic content. This increase results from a structural release mechanism. In raw plant tissue, a significant portion of polyphenols is covalently bound to cell wall polysaccharides (hemicellulose, pectin, cellulose) via ester and ether bonds, making them difficult to digest in this form (65). However, technologies such as HPP and US physically disrupt cell compartments through the high shear forces and pressure shocks they impose on tissue. Thus, phenolic compounds detach from the polysaccharide network, and ester bonds are hydrolyzed. Consequently, bound phenolics trapped in the matrix are released as free phenolics with greater water solubility and intestinal absorption, ultimately contributing to increased antioxidant capacity. However, the balance between enhanced phenolic release and potential oxidative degradation must be carefully managed. For example, the US can generate ROS during acoustic cavitation, which may oxidize the newly released free phenolics if processing parameters are not rigorously optimized (52).

Furthermore, evaluating the antioxidant properties of these foods requires a critical approach to analytical methodologies. Currently, antioxidant capacity is predominantly measured using *in vitro* spectrophotometric assays (such as DPPH, ABTS, and FRAP). While these methods are cost-effective and provide rapid baseline data regarding free radical scavenging, they do not account for physiological conditions such as cellular uptake or bioavailability. Cellular models, such as assessing intracellular ROS inhibition, offer a more biologically relevant perspective but still lack the complexity of systemic human metabolism. This methodological gap underscores the critical need for human clinical trials to validate whether the enhanced *in vitro* bioactivity translates to tangible health benefits *in vivo*.

The protective effect of non-thermal technologies is particularly evident in their ability to maintain pigment integrity and stabilize product color by effectively suppressing oxidative enzyme activity (66). Browning or olive-green discoloration, caused by the loss of the magnesium atom at the center of chlorophyll during traditional thermal processing, is largely prevented by HPP and PEF applications, which do not affect covalent bonds, thereby maintaining the bright green color of the fresh product (67). The situation is even more advantageous regarding carotenoids (lutein, β-carotene). While non-thermal processing increases the release of these lipophilic components by permeabilizing cell membranes, it can limit the conversion of the trans-form to the cis-form, a process common in thermal processing and negatively affecting bioavailability (68).

As a result, Total Antioxidant Capacity (TAC), representing the food’s power to neutralize free radicals, is measured at levels equivalent to, or sometimes higher than, the fresh product in GLVs juices treated with these technologies (53). Methods such as US, CP, and PEF not only preserve existing antioxidants but also activate biosynthesis pathways by creating controlled abiotic stress in the tissue. These technologies significantly increase antioxidant capacity (DPPH, ABTS) compared to thermal processing by releasing bound phenolics through cell wall modification and inactivating oxidative enzymes. These findings confirm the potential of non-thermal processing, defined as functional enhancement (69). In general, although traditional thermal pasteurization ensures microbial safety, it causes serious losses of heat-sensitive components such as vitamin C and glucosinolates. In contrast, non-thermal technologies offering cold pasteurization minimize these losses. However, when enzymes such as PPO and POD cannot be fully inactivated, quality losses may occur during storage. Therefore, the current approach highlights combined hurdle technologies, such as thermosonication (mild heat + US), where low temperature and physical methods are integrated, as the most effective strategy (51).

## 7 Bioaccessibility and bioavailability of bioactive components

The effects of polyphenols on human health depend on absorption capacity, which is generally low and highly variable among individuals. In this context, three fundamental concepts stand out (70). The first stage, bioaccessibility, refers to the proportion of a compound ingested that is liberated from the food matrix and becomes available for absorption in the intestine during digestion. This process is not limited to the release of the component; it also encompasses metabolic interactions, such as polyphenols regulating glucose and lipid release by inhibiting digestive enzymes (71). Bioavailability, the second stage, is the portion of this released fraction that crosses the intestinal barrier, enters systemic circulation, and reaches target tissues (72). However, since polyphenols in foods are generally found in polymeric or glycosylated forms, they require transformation for absorption. In this process, called biotransformation, compounds are hydrolyzed by intestinal enzymes or colonic microflora; the resulting metabolites generally have a much higher absorption potential than the native compounds in the original food (70).

*In vitro* digestion models are widely preferred for understanding the behavior of foods in the human gastrointestinal system and for tracking the fate of bioactive components, owing to ethical constraints and cost advantages. For GLVs and their products, the INFOGEST static *in vitro* digestion model is one of the most widely used and standardized methods. This protocol standardizes physiological conditions (pH, temperature, enzyme activities, bile salts, and mechanical forces) in the oral, gastric, and small intestinal phases, ensuring comparability of results among researchers (73). The INFOGEST model is highly effective in determining the release kinetics and chemical stability of polyphenols and vitamins during digestion. In recent years, semi-dynamic models have been proposed to overcome the limitations of static models and more realistically simulate processes such as gastric emptying and instantaneous pH changes (73, 74).

Although the INFOGEST protocol provides a highly standardized *in vitro* digestion model (75, 76). Significant methodological gaps remain. Primarily, the methods for treating the digesta post-digestion (e.g., centrifugation parameters, extraction solvents) lack standardization, making inter-study comparisons difficult. Furthermore, there is a critical shortage of studies reporting the bioaccessibility of specific compounds using advanced chromatographic techniques (e.g., HPLC or LC–MS). Many existing studies rely on spectrophotometric methods, which are highly susceptible to matrix interferences. In the context of non-thermal processing, the process-matrix-bioactive interaction requires deeper mechanistic investigation. For instance, recent literature highlights that US processing significantly enhances the bioaccessibility of phenolic compounds and the antioxidant capacity of selected vegetables (77). However, translating these *in vitro* extraction efficiencies into predictive models of human digestion remains a vital opportunity for future research.

The scope of different simulation models used in the literature and gaps regarding the clinical validity of *in vitro* findings, in particular, are summarized in Table 3.

**Table 3:** Assessment of bioaccessibility and bioavailability of GLVs bioactives.

Model type	Metric/focus	Expected outcomes and research implications	Critical limitation/gap	References
Elderly simulation (*In Vitro*)	Age-related digestion: Absorption under reduced acid/enzyme conditions	Increase (+): Matrices homogenized by HPP increase antioxidant absorption despite low digestive capacity in the elderly	Clinical validation is lacking; it relies solely on chemical simulation in a laboratory setting	(92)
Dynamic models	Process kinetics: Physical/chemical changes during digestion	Detailed analysis: Mimics food breakdown mechanics and nutrient release more realistically	Complex setup and costly; data in the literature is scarce	(73)
*In Vivo*	Bioavailability: Transfer to systemic circulation	Insufficient data: Evidence regarding the reflection of increased bioaccessibility on blood metabolite levels is limited	Most current studies remain at the *in vitro* stage; human trials are lacking	(70)

HPP, High Hydrostatic Pressure, US, Ultrasound; PEF, Pulsed Electric Fields; CP, Cold Plasma.

**Figure 2:**
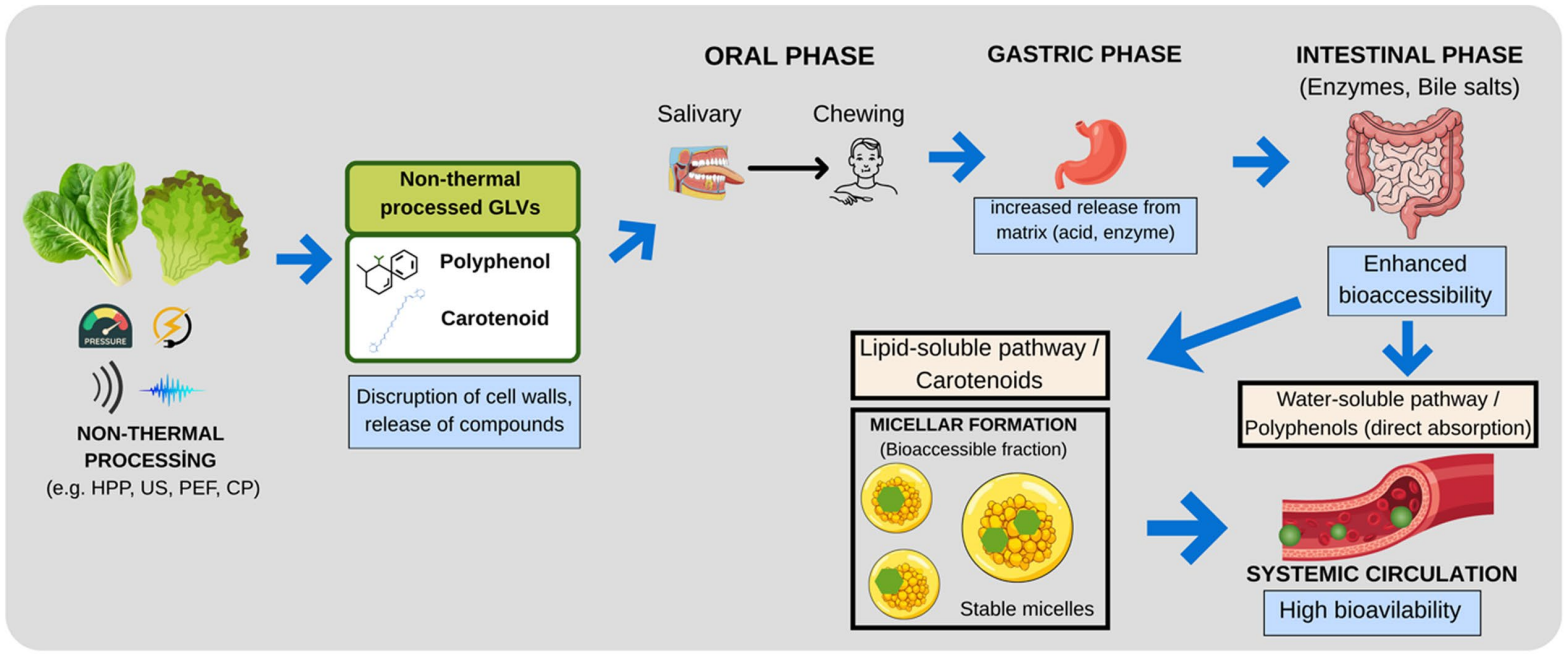
Impact of non-thermal processing on the gastrointestinal fate and bioavailability of GLVs bioactives. HPP, High Pressure Processing; PEF, Pulsed Electric Fields; CP, Cold Plasma; US, Ultrasound.

One of the most critical factors determining digestibility and absorption is the composition of the food matrix. For instance, proteins form complexes with anthocyanins through hydrophobic interactions and hydrogen bonds, protecting these sensitive compounds against environmental factors and potentially preventing their degradation, especially in the acidic environment of the stomach (78, 79). However, the most complex mechanism in matrix interactions involves dietary fibers. Fibers can slow digestion by binding with polyphenols or blocking the active sites of digestive enzymes (particularly α-amylase and lipase). Although the entrapment of polyphenols by fibers acts as a physical barrier that limits rapid absorption in the small intestine, this can actually create an advantage. Following gastrointestinal release, lipophilic bioactives such as carotenoids must be incorporated into mixed micelles—composed of bile salts, lipid digestion products, and cholesterol—to become bioaccessible. This micellar formation is a critical step that facilitates the transport of these compounds across the unstirred water layer of the enterocytes for subsequent intestinal absorption. Protected polyphenols are transported intact to the colon, where they are fermented by the microbiota, increasing the production of valuable “post-biotic” metabolites with systemic effects (80). The contribution of applied processing techniques to the bioavailable fraction, achieved by maintaining the gastrointestinal stability of these bioactives, is illustrated in the model presented in Figure 2.

## 8 Implications of non-thermal technologies for product design, sensory aspects, and shelf life

Matrix softening and bioactive component degradation caused by traditional thermal processing conflict with consumer demand for minimally processed food. Therefore, the industrial trend has shifted toward technologies such as HPP and CP, which ensure food safety without applying thermal processing (20). These technologies, which retain fresh-like sensory properties by preserving cell wall integrity, also extend shelf life within safe limits by achieving microbial inactivation. This approach paves the way for the development of functional beverages and snacks that are both microbiologically safe and nutritionally preserved, particularly for elderly consumers with compromised immune systems (81, 111). The efficacy of the selected technology depends largely on the physical form (matrix structure) of the processed vegetable; specific quality effects in different matrices (fresh leaf, puree, powder) are detailed in Table 4.

**Table 4:** Technology and outcome impact matrix in GLVs.

Vegetable matrix	Technology and parameters	Target outcome (bioactive/quality)	Enzymatic and microbial effect	Sensory and physical properties	References
Spinach (fresh)	DBD gas: atmospheric air	Following the CPPE application (10 min) and 7 days of storage, significant increases were observed in pH and weight loss data	4.6 ± 0.6, 4.8 ± 1.7 log reduction in *E. coli* and *L. innocua*	Observation of surface browning in spinach leaves	(107)
Wheatgrass (juice)	HPP: 500 MPa for 60 s	Increased the chlorophyll content by 9.0%	25.9% reduction in POD level	No significant changes in color parameters	(108)
Smoothie/vegetable juice (mixed GLVs)	PEF: 10–35 kV/cm, 1–50 μs pulses	Intracellular polyphenol extraction increases; Total Antioxidant Capacity (TAC) increases	Pasteurization effect on vegetative microorganisms (requires cold chain)	Viscosity modification; Fresh-like sensory properties	(86)
Rocket	Cold atmospheric dielectric barrier discharge plasma	Significant decrease in pH	1.020, 0.298, 0.493, and 0.996 log CFU/g reduction for the Total Viable Flora, *Pseudomonas* spp., yeasts/molds, and the lactic acid bacteria	No significant changes in color and hardness parameters	(109)
Rocket	HP 400 Mpa 2 min	No significant change in the amounts of Cr, Cu, Fe, Mn, Ni, and Zn	1.5–2.0 log reduction in aerobic mesophilic bacteria level	Increasing in sweetness, acidity, bitterness, saltiness, and aroma intensity values	(110)

HP, High Pressure; HPP, High Hydrostatic Pressure; US, Ultrasound; PEF, Pulsed Electric Fields; CP, Cold Plasma; DBD, Dielectric Barrier Discharge; IDDSI, International Dysphagia Diet Standardization Initiative; CPPE, Complicated parapneumonic effusion.

When looking at product categories, browning and microbial load increase, which are the biggest problems in fresh-cut salads, can now be managed with chlorine-free green technologies. For example, Dielectric Barrier Discharge (DBD) plasma applications reduce the pathogen (*Escherichia coli*, Listeria) load on lettuce and spinach surfaces by 2–3 logs while preserving tissue hardness and chlorophyll content (82). Regarding US applications, US-assisted washing protocols, in particular, ensure the physical detachment of microorganisms from the plant tissue surface through the acoustic cavitation they generate (83). It has been reported that when this process is combined with MAP technology in a synergistic approach, the microbiological quality of the products is preserved, and shelf life can be extended up to a maximum of 14 to 21 days for fresh-cut lettuce and spinach (84, 85). Non-thermal technologies offer significant advantages not only for solid matrices, such as fresh-cut salads, but also for liquid-based formulations. In liquid functional beverages (smoothies, fruit juices), controlling oxidative enzymes is a priority. HPP technology, which applies 400–600 MPa pressure, inactivates PPO and POD enzymes and preserves over 90% of vitamin C and chlorophyll; this ensures the stability of the product’s bright green color (67). PEF, which creates electroporation in the cell membrane, facilitates the extraction of intracellular polyphenols into the liquid phase. This permeabilization effect increases the antioxidant capacity of the final product, offering a more intense bioactive profile for elderly consumers (86). In addition to keeping food and drink nutritious, these technologies can also be used to alter the structure of thicker products designed to meet specific medical requirements. Viscosity control is of vital importance in GLVs-based purees and cold soups developed for the management of dysphagia (swallowing difficulty), which is frequently seen in elderly individuals. US application optimizes product consistency (viscosity) by affecting pectin methylesterase enzyme activity and reducing particle size. This modification has been shown to prevent phase separation and create a homogeneous structure suitable for safe swallowing by the elderly (compliant with IDDSI standards) (87).

The versatility of non-thermal processing is also evident in the production of powdered derivatives, for which structural integrity is essential for efficiency. In powder products, PEF applied before drying loosens the cell structure and accelerates water diffusion, thereby helping preserve heat-sensitive glucosinolates by reducing drying time and heat exposure ((88); Sarkar et al., 2023). Regarding microbial safety, although non-thermal technologies meet Food and Drug Administration (FDA) standards (5-log reduction) for vegetative pathogens, the cold chain requirement persists because they are limited in effectiveness against bacterial spores (89). It is reported that the Hurdle Technology approach, developed to overcome this challenge and in which physical methods are supported by natural antimicrobials, can significantly extend the refrigerated shelf life of GLVs juices while preserving their sensory quality (90). Consumer research indicates that non-thermally processed GLVs products are preferred by the health-conscious elderly population due to their fresh, natural perception; however, food technology neophobia and price can pose barriers (91). In sensory panel tests, products processed with HPP exhibited a more vivid green color and a fresher aroma profile than their thermally processed counterparts (67). Especially in the fight against inflammaging, GLVs products processed with these technologies are strategic for increasing the bioaccessibility of carotenoids (lutein, β-carotene). *In vitro* models that simulate the digestive conditions of elderly individuals (reduced gastric acid and enzyme secretion) confirm that HPP-processed vegetable matrices significantly increase antioxidant absorption despite these physiological limitations (92).

## 9 Knowledge gaps, challenges, and future perspectives

While the success of promising non-thermal technologies for processing GLVs in laboratory settings is indisputable, the biggest obstacle facing these methods is the lack of standardization of HPP, PEF, and CP parameters, as well as yield losses experienced during the transition to industrial scale (89). To overcome these barriers to commercialization, engineering constraints specific to each technology must be resolved. For instance, the batch-operation requirement of most HPP systems increases costs by extending product loading and unloading times; the solution to this problem lies in the evolution of industrial designs toward semi-continuous systems, especially for liquid products (54, 88). In PEF applications, non-homogeneous distribution of the electric field in large treatment chambers creates the risk of “hot spots” or insufficient treatment; however, it is predicted that this problem can be overcome by improving the treatment chamber geometry and integrating Artificial Intelligence (AI)-supported simulations into the process (56, 86, 93). Similarly, in US technology, the energy loss as sound waves propagate to the center of large-volume tanks reduces the process’s effectiveness. To increase energy efficiency, using multiple transducers placed at different points in the reactor, rather than a single source, is the most effective approach for maximizing cavitation efficiency (51, 83). In CP management, the limited penetration depth of plasma technology limits access to the inner surfaces of bulk leaves; to overcome this, dynamic tunnel systems in which products are continuously moved on vibrating belts to expose all surfaces to the gas should be developed (94).

In the food industry, traditional trial-and-error methods remain inefficient, especially for complex multi-parameter systems such as HPP and CP. At this point, AI and Machine Learning (ML) algorithms reduce costs by providing real-time optimization of critical parameters such as pressure, frequency, and processing time (93). ML-based prediction models and the “Digital Twin” approach accelerate the industry’s transition to smart production lines by predicting nutrient losses and shelf life in sensitive matrices like GLVs with high accuracy (95). Furthermore, current Deep Learning (DL) algorithms working at the molecular level can simulate polyphenol-protein interactions *in silico* (computer environment), while AI-supported sensors proactively detect microbial risks, increasing traceability in the fresh produce chain (96). On the analytical side, traditional methods are insufficient to explain complex molecular changes. Simply measuring total phenolic content is no longer adequate; at this point, the “Foodomics” approach, combining genomic and metabolomic data, offers a new holistic standard for understanding food safety and bioactivity (97). Future vision should focus on multi-omics models that match chemical changes in food with biological responses in the human body (94).

From a sustainability perspective, non-thermal processing technologies offer production models that align with the UN Sustainable Development Goals (SDGs) by reducing energy and water consumption. Life Cycle Assessment (LCA) studies confirm that these methods reduce carbon footprints, but high initial Capital Expenditures (CAPEX) remain a significant barrier to industrial integration (98). Despite this, the growing consumer demand for “clean labels” and the potential integration of these technologies into circular economy models, where food waste is valorized, are increasing their market share. The ultimate goal is to use technology not just for shelf life, but for biological impact. GLVs play a key role in healthy aging by feeding the gut microbiota with their polyphenols; however, reduced digestive capacity in elderly individuals hinders absorption (99). Technologies like HPP and US can facilitate the production of beneficial metabolites, even in individuals with low digestive capacity, by opening the plant matrix and increasing bioaccessibility. Therefore, future research should focus on healthy aging strategies by providing clinical evidence on how technology supports the microbiota (100).

## 10 Conclusion

This review demonstrates that non-thermal processing technologies such as HPP, PEF, US, and CP offer a superior profile compared to traditional thermal processing for preserving and enhancing the functional potential of GLVs. Reviewed studies indicate that these technologies not only ensure food safety but also facilitate the release of matrix-bound polyphenols and carotenoids by inducing controlled physical modifications in plant cell walls. Particularly considering the reduced digestive capacity and inflammaging risk associated with aging, these new-generation foods—with increased bioaccessibility, preserved antioxidant capacity, and sensory properties close to those of the fresh product—serve as a critical tool for preventive medicine and personalized nutrition strategies. From a technological perspective, although it has been proven that HPP and PEF applications extend shelf life by preserving the chemical stability of heat-sensitive vitamins and pigments, the inability to fully inactivate enzymes (especially PPO and POD) at the industrial scale and quality losses during storage remain challenges to overcome. Furthermore, the fact that a large portion of existing data relies on static *in vitro* digestion models raises the question of whether the observed laboratory increases in bioaccessibility translate into a clinically meaningful biological response in human metabolism. From a future perspective, the success of non-thermal technologies depends on the standardization of engineering parameters and the development of hybrid approaches that will increase process efficiency. The research vision should move beyond technological optimization and focus on clinical studies examining the long-term effects of these products on the gut microbiota and oxidative stress markers of the elderly population. In conclusion, these technologies, integrated with AI-supported modeling and foodomics approaches, are positioned to play a decisive role in the rational design of sustainable, safe, and high-bioavailability foods.

## References

[1] WHO. Ageing and Health: Global Population Trends 2024–2030. WHO Press (Switzerland: World Health Organization Press) (2025).

[2] Jiang B Dong YN Xiong Y Jiang CX Ping J Wu Q . Global research trends in inflammaging from 2005 to 2024: a bibliometric analysis. Front Aging. (2025) 6:1554186. doi: 10.3389/fragi.2025.1554186, 40276724 PMC12018403

[3] Abadir PM Bandeen-Roche K Bergeman C Bennett D Davis D Kind A . An overview of the resilience world: proceedings of the American Geriatrics Society and National Institute on Aging state of resilience science conference. J Am Geriatr Soc. (2023) 71:2381–92. doi: 10.1111/jgs.18388, 37079440 PMC10523918

[4] Rovira II Biragyn A Brown LL Galis ZS Klauzinska M Kotliarova SE . Health and aging trajectories: shared and competing risks and resiliencies for chronic diseases associated with aging. A NIH-wide workshop. Front Public Health. (2025) 13:1462217. doi: 10.3389/fpubh.2025.1462217, 40376061 PMC12078139

[5] Polom J Boccardi V. Employing nutrition to delay aging: a plant-based telomere-friendly dietary revolution. Nutrients. (2025) 17:2004. doi: 10.3390/nu17122004, 40573115 PMC12196515

[6] Salminen A Kaarniranta K Kauppinen A. Inflammaging: disturbed interplay between autophagy and inflammasomes. Aging. (2012) 4:166–75. doi: 10.18632/aging.100444, 22411934 PMC3348477

[7] Liu Y Wang R Liu H Tu Z. Dietary phytochemicals targeting NRF2 against skin cellular senescence: mechanistic insights and potential for functional food development. Biology. (2025) 15:39. doi: 10.3390/biology15010039, 41514880 PMC12785134

[8] Das G Kameswaran S Ramesh B Bangeppagari M Nath R Das Talukdar A . Anti-aging effect of traditional plant-based food: an overview. Foods. (2024) 13:3785. doi: 10.3390/foods13233785, 39682858 PMC11639806

[9] Davinelli S Medoro A Hu FB Scapagnini G. Dietary polyphenols as geroprotective compounds: from blue zones to hallmarks of ageing. Ageing Res Rev. (2025) 108:102733. doi: 10.1016/j.arr.2025.102733, 40120947

[10] Kumkum R Aston-Mourney K McNeill BA Hernández D Rivera LR. Bioavailability of anthocyanins: whole foods versus extracts. Nutrients. (2024) 16:1403. doi: 10.3390/nu16101403, 38794640 PMC11123854

[11] Landry MJ Ward CP. Health benefits of a plant-based dietary pattern and implementation in healthcare and clinical practice. Am J Lifestyle Med. (2024) 18:657–65. doi: 10.1177/15598276241237766, 39309320 PMC11412377

[12] Fatima G Dalmadi I Süllős G Takács K Halmy E. Dietary patterns for health-span and longevity: a comprehensive review of nutritional strategies promoting lifelong wellness. Appl Sci (Switz). (2025) 15:12013. doi: 10.3390/app152212013

[13] Kariminezhad Z Rahimi M Fernandes J Fahmi H Benderdour M. Protective effect of resolvin D1, D2, and their methyl esters on oxidative stress and hyaluronidase—induced hyaluronic acid degradation. Antioxidants. (2026) 15:163. doi: 10.3390/antiox15020163, 41750543 PMC12937457

[14] Moni SS. "An overview of natural antioxidant pharmacodynamics and pharmacokinetics". In: Recent Advances in Oxidative Stress Associated Chronic Diseases Volume 2: A Review of The Health Benefits and Risks of The Substance (2026). p. 361–401. Springer Nature.

[15] El-Saadony MT Saad AM Mohammed DM Alkafaas SS Abd El-Mageed TA Fahmy MA . Plant bioactive compounds: extraction, biological activities, immunological, nutritional aspects, food application, and human health benefits—a comprehensive review. Front Nutr. (2025) 12:1659743. doi: 10.3389/fnut.2025.1659743, 41487672 PMC12757306

[16] Thakur V Mal D Soga K Gandhi A. A review on nutritional quality of green leafy vegetables. Ecol Environ Conserv. (2022) 28, S351–6. doi: 10.53550/EEC.2022.v28i06s.059

[17] Tkaczenko H Buyun L Kołodziejska R Kamiński P Kurhaluk N. Neuroactive phytochemicals as multi-target modulators of mental health and cognitive function: an integrative review. Int J Mol Sci. (2025) 26:8907. doi: 10.3390/ijms26188907 41009475 PMC12469506

[18] Adedeji FT Adeyanju AA Bamidele OP. Phenolic profile, physicochemical properties and antioxidant activities of blanching water obtained from some green leafy vegetables. Food Chem Adv. (2025) 6:100883. doi: 10.1016/j.focha.2024.100883

[19] Barbhuiya RI Singha P Singh SK. A comprehensive review on impact of non-thermal processing on the structural changes of food components. Food Res Int. (2021) 149:110647. doi: 10.1016/j.foodres.2021.110647, 34600649

[20] Ağagündüz D Ayakdaş G Katırcıoğlu B Ozogul F. Advances in non-thermal food processing: a comprehensive approach to nutrient retention, food quality, and safety. Sustain Food Technol. (2025) 3:1284–308. doi: 10.1039/D5FB00136F

[21] Özdemir E Başaran P Kartal S Akan T. Cold plasma application to fresh green leafy vegetables: impact on microbiology and product quality. Compr Rev Food Sci Food Saf. (2023) 22:4484–515. doi: 10.1111/1541-4337.13231, 37661766

[22] Farrokhi F Badii F Ehsani MR Hashemi M. Ultrasound treatment of preformed nanofibrillated whey protein isolate: effects on structure, enzymatic hydrolysis, and bioactivity. Food Hydrocoll Health. (2026) 9:100267. doi: 10.1016/j.fhfh.2026.100267, 23194546

[23] Naseem T Altemimi AB Ali K Zahid N Fatima S Naeem M . Exploring the potential of emerging thermal and non-thermal processing techniques for tomato juice preservation: a comprehensive review. Future Foods. (2026) 13:100956. doi: 10.1016/j.fufo.2026.100956

[24] Quintero-Quiroz J Zuluaga-Arroyave N Valencia-Naranajo A Molina-Castillo MC Varela-Garcia N Medina-Rodriguez M . Non-thermal hydrodynamic cavitation for surplus fruits and vegetables: improved vitamin C and bioactive preservation. Foods. (2026) 15:268. doi: 10.3390/foods15020268 41596867 PMC12839826

[25] Zhu Z Huang X Ma G Wan F Zang ZP Dai F . Optimization of *Scutellaria baicalensis* drying using combined ultrasound and vacuum far-infrared technology: drying kinetics and bioactive preservation. Food Bioprocess Technol. (2025) 19:88. doi: 10.1007/s11947-025-04137-5

[26] Rabeh K Hnini M Oubohssaine M. A comprehensive review of transcription factor-mediated regulation of secondary metabolites in plants under environmental stress. Stress Biol. (2025) 5:15. doi: 10.1007/s44154-024-00201-w

[27] Nurzyńska-Wierdak R. Green leafy vegetables (GLVs) as nutritional and preventive agents supporting metabolism. Meta. (2025) 15:502. doi: 10.3390/metabo15080502, 40863121 PMC12388681

[28] Sarma U Bhavya TR. Dietary phytonutrients in common green leafy vegetables and the significant role of processing techniques on spinach: a review. Food Prod Process Nutr. (2024) 6:10. doi: 10.1186/s43014-023-00192-7

[29] Knez M Mattas K Gurinovic M Gkotzamani A Koukounaras A. Revealing the power of green leafy vegetables: cultivating diversity for health, environmental benefits, and sustainability. Glob Food Sec. (2024) 43:100816. doi: 10.1016/j.gfs.2024.100816

[30] Karadağ G Karaman AD Öğüt S. Meyve ve sebzelerde bulunan biyoaktif bileşenlerin sağlık üzerine etkileri. Toros Univ J Food Nutr Gastron. (2022) 1:77–90. doi: 10.58625/jfng-1837

[31] Saini RK Song MH Yu JW Lee JH Ahn HY Keum YS . Profiling of nutritionally vital bioactive compounds in emerging green leafy vegetables: a comparative study. Foods. (2022) 11:3867. doi: 10.3390/foods11233867, 36496677 PMC9736515

[32] Arslan E Günal AM. Lutein ve zeaksantin. Sağlık ve Yaşam Bilimleri Dergisi. (2022) 4:201–8. doi: 10.33308/2687248X.202241233

[33] NavaneethaKrishnan S Rosales JL Lee KY. ROS-mediated cancer cell killing through dietary phytochemicals. Oxidative Med Cell Longev. (2019) 2019:9051542. doi: 10.1155/2019/9051542, 31217841 PMC6536988

[34] Valdez-Miranda JI Guitiérrez-López GF de la Robles-Torre RR Hernández-Sánchez H Robles-López MR. Health benefits of high voltage electrostatic field processing of fruits and vegetables. Plant Foods Hum Nutr. (2024) 79:260–9. doi: 10.1007/s11130-024-01190-x, 38761282

[35] Bondonno CP Dalgaard F Blekkenhorst LC Murray K Lewis JR Croft KD . Vegetable nitrate intake, blood pressure and incident cardiovascular disease: Danish diet, Cancer, and health study. Eur J Epidemiol. (2021) 36:813–25. doi: 10.1007/s10654-021-00747-3, 33884541 PMC8416839

[36] Holland TM Agarwal P Wang Y Dhana K Leurgans SE Shea K . Association of Dietary Intake of Flavonols with changes in global cognition and several cognitive abilities. Neurology. (2023) 100:e694–702. doi: 10.1212/WNL.0000000000201541, 36414424 PMC9969915

[37] Çınar EN Eravcı H Öksüz M Toprak K. Beslenme ve Cilt Sağlığı İlişkisinde Fitokimyasalların Yeri: Biyolojik Mekanizmalar ve Terapötik Etkiler. Mersin Üniversitesi Tıp Fakültesi Lokman Hekim Tıp Tarihi ve Folklorik Tıp Dergisi. (2025) 15:828–38. doi: 10.31020/mutftd.1625797

[38] Hu FB. Diet strategies for promoting healthy aging and longevity: an epidemiological perspective. J Intern Med. (2024) 295:508–31. doi: 10.1111/joim.13728, 37867396 PMC10939982

[39] Sanatombi K. Antioxidant potential and factors influencing the content of antioxidant compounds of pepper: a review with current knowledge. Compr Rev Food Sci Food Saf. (2023) 22:3011–52. doi: 10.1111/1541-4337.13170, 37184378

[40] Lugumira R Tafiire H Vancoillie F Ssepuuya G Van Loey A. Nutrient and phytochemical composition of nine African leafy vegetables: a comparative study. Foods. (2025) 14:1304. doi: 10.3390/foods14081304, 40282706 PMC12027459

[41] Yang J Luo J Tian X Zhao Y Li Y Wu X. Progress in understanding oxidative stress, aging, and aging-related diseases. Antioxidants. (2024) 13:394. doi: 10.3390/antiox13040394, 38671842 PMC11047596

[42] Sarak E Ceylan Y. Mitokondriyal Yaşlanma. Bartın Univ Int J Nat Appl Sci. (2025) 8:293–307. doi: 10.55930/jonas.1741124

[43] Singh A Schurman SH Bektas A Kaileh M Roy R Wilson DM . Aging and inflammation. Cold Spring Harb Perspect Med. (2024) 14:a041197. doi: 10.1101/cshperspect.a041197, 38052484 PMC11146314

[44] Lee CH Lee SH. Research progress on anti-aging with natural products: from pathway modulation to AI-driven discovery. Biomolecules. (2025) 15:1384. doi: 10.3390/biom15101384, 41154613 PMC12564003

[45] Jacquier EF Kassis A Marcu D Contractor N Hong J Hu C . Phytonutrients in the promotion of healthspan: a new perspective. Front Nutr. (2024) 11:1409339. doi: 10.3389/fnut.2024.1409339, 39070259 PMC11272662

[46] Lohana P Suryaprawira A Woods EL Dally J Gait-Carr E Alaidaroos NYA . Role of Enzymic antioxidants in mediating oxidative stress and contrasting wound healing capabilities in Oral mucosal/skin fibroblasts and tissues. Antioxidants. (2023) 12:1374. doi: 10.3390/antiox12071374, 37507914 PMC10375950

[47] Hao M Ding C Peng X Chen H Dong L Zhang Y . Ginseng under forest exerts stronger anti-aging effects compared to garden ginseng probably via regulating PI3K/AKT/mTOR pathway, SIRT1/NF-κB pathway and intestinal flora. Phytomedicine. (2022) 105:154365. doi: 10.1016/j.phymed.2022.154365, 35930860

[48] Mahto K Kuwar OK Maloo A Kalia N. Therapeutic potential of luteolin in neurodegenerative disorders: targeting Nrf2, NFĸB, MAPK, and JAK-STAT pathways to combat neuroinflammation and apoptosis. Inflammopharmacology. (2025) 33:5011–21. doi: 10.1007/s10787-025-01846-3, 40694206

[49] Debnath I Ghosh S Jha SK Bhunia S Nayak A Basak S . Mechanistic insights and therapeutic potential of quercetin in neuroprotection: a comprehensive review of pathways and clinical perspectives. BIO Integr. (2025) 6, 1–31. doi: 10.15212/bioi-2025-0073

[50] Chakka AK Sriraksha MS Ravishankar CN. Sustainability of emerging green non-thermal technologies in the food industry with food safety perspective: a review. LWT. (2021) 151:112140. doi: 10.1016/j.lwt.2021.112140

[51] Bermudez-Aguirre D Niemira BA. Pasteurization of foods with ultrasound: the present and the future. Appl Sci. (2022) 12:10416. doi: 10.3390/app122010416

[52] Abdulstar AR Altemimi AB Al-Hilphy AR. Exploring the power of Thermosonication: a comprehensive review of its applications and impact in the food industry. Foods. (2023) 12:1–22. doi: 10.3390/foods12071459, 37048278 PMC10094072

[53] Yıkmış S Türkol M Dülger Altıner D Duman Altan A Sağlam K Abdi G . Advancing sustainable food preservation: ultrasound and thermosonication as novel approaches to enhance nutritional and bioactive properties of broccoli juice. Food Chem X. (2025) 27:102412. doi: 10.1016/j.fochx.2025.102412, 40231120 PMC11995051

[54] Nabi BG Mukhtar K Arshad RN Radicetti E Tedeschi P Shahbaz MU . High-pressure processing for sustainable food supply. Sustainability. (2021) 13:13908. doi: 10.3390/su132413908

[55] Westphal A Schwarzenbolz U Böhm V. Effects of high pressure processing on bioactive compounds in spinach and rosehip puree. Eur Food Res Technol. (2018) 244:395–407. doi: 10.1007/s00217-017-2964-5

[56] Landi G Benedetti M Sforzini M Eslami E Pataro G. Comparative analysis of cost, energy efficiency, and environmental impact of pulsed electric fields and conventional thermal treatment with integrated heat recovery for fruit juice pasteurization. Foods. (2025) 14:2239. doi: 10.3390/foods14132239, 40646991 PMC12248458

[57] Bocker R Silva EK. Pulsed electric field technology as a promising pre-treatment for enhancing orange agro-industrial waste biorefinery. RSC Adv. (2024) 14:2116–33. doi: 10.1039/d3ra07848e, 38196909 PMC10775899

[58] Perner J Matoušek J Auer Malinská H. Cold plasma treatment influences the physiological parameters of millet. Photosynthetica. (2024) 62:126–37. doi: 10.32615/ps.2024.010, 39650629 PMC11609773

[59] Gutiérrez DR Rodríguez SDC. Combined effect of UV-C and ozone on bioactive compounds and microbiological quality of fresh-cut rocket leaves. Am J Food Sci Technol. (2019) 7:71–8. doi: 10.12691/ajfst-7-3-1

[60] Mmbando GS. Harnessing UV radiation for enhanced agricultural production: benefits on nutrition, quality, and sustainability. All Life. (2024) 17:2381141. doi: 10.1080/26895293.2024.2381141

[61] Boateng ID. Recent processing of fruits and vegetables using emerging thermal and non-thermal technologies. A critical review of their potentialities and limitations on bioactives, structure, and drying performance. Crit Rev Food Sci Nutr. (2024) 64:4240–74. doi: 10.1080/10408398.2022.2140121, 36315036

[62] Rojas ML Trevilin JH Funcia E d S Gut JAW Augusto PED. Using ultrasound technology for the inactivation and thermal sensitization of peroxidase in green coconut water. Ultrason Sonochem. (2017) 36:173–81. doi: 10.1016/j.ultsonch.2016.11.028, 28069198

[63] Arshad RN Abdul-Malek Z Roobab U Munir MA Naderipour A Qureshi MI . Pulsed electric field: a potential alternative towards a sustainable food processing. Trends Food Sci Technol. (2021) 111:43–54. doi: 10.1016/j.tifs.2021.02.041

[64] Nawaz A Irshad S Luo X Walayat N Harlina PW Qin Z . Advances in green food processing technologies: enhancing efficiency, safety, and sustainability. Food Control. (2026) 181:111715. doi: 10.1016/j.foodcont.2025.111715

[65] Jia H Ren F Liu H. Effects and improvements of storage conditions and processing on the bioaccessibility and bioavailability of phytochemicals in fruits and vegetables. Int J Food Sci Technol. (2025) 60:vvae040. doi: 10.1093/ijfood/vvae040

[66] Yang R Huang S Wang W Sun T Ai M Han X . Effects of thermal and non-thermal sterilization on the quality and flavor of prune jam: an integrated analysis using E-nose, GC–MS, and GC-IMS. Food Res Int. (2026) 230:118656. doi: 10.1016/j.foodres.2026.118656 41794510

[67] Pérez-Lamela C Torrado-Agrasar AM. Effects of high-pressure processing (HPP) on antioxidant vitamins (a, C, and E) and antioxidant activity in fruit and vegetable preparations: a review. Appl Sci. (2025) 15:10699. doi: 10.3390/app151910699

[68] Kim SY Lee BM Hong SY Yeo HH Jeong SH Lee DU. A pulsed electric field accelerates the mass transfer during the convective drying of carrots: drying and rehydration kinetics, texture, and carotenoid content. Foods. (2023) 12:589. doi: 10.3390/foods12030589, 36766117 PMC9914679

[69] Umair M Abid M Mumraiz M Jabbar S Xun S Ameer K . Emerging frontiers in juice processing: the role of ultrasonication and other non-thermal technologies in enhancing antioxidant capacity and quality of fruit and vegetable juices. Ultrason Sonochem. (2025) 122:107554. doi: 10.1016/j.ultsonch.2025.107554, 41016255 PMC12510019

[70] Polia F Pastor-Belda M Martínez-Blázquez A Horcajada M-N Tomás-Barberán FA García-Villalba R. Technological and biotechnological processes to enhance the bioavailability of dietary (poly)phenols in humans. J Agric Food Chem. (2022) 70:2092–107. doi: 10.1021/acs.jafc.1c07198, 35156799 PMC8880379

[71] Sharma V Devkota L Kishore N Dhital S. Understanding the interplay between dietary fiber, polyphenols, and digestive enzymes. Food Hydrocoll. (2025) 166:111310. doi: 10.1016/j.foodhyd.2025.111310

[72] Grundy MML Moughan PJ Wilde PJ. Bioaccessibility and associated concepts: need for a consensus. Trends Food Sci Technol. (2024) 145:104373. doi: 10.1016/j.tifs.2024.104373

[73] Zhou H Tan Y McClements DJ. Applications of the INFOGEST *in vitro* digestion model to foods: a review. Annu Rev Food Sci Technol. (2023) 14:135–56. doi: 10.1146/annurev-food-060721-012235, 36446138

[74] Morales D Iriondo-DeHond A Fernández-Tomé S. Application of the INFOGEST 2.0 standardized method to study the behavior of phenolic compounds throughout gastrointestinal digestion. Food Chem. (2025) 492:145531. doi: 10.1016/j.foodchem.2025.145531, 40675074

[75] Brodkorb A Egger L Alminger M Alvito P Assunção R Ballance S . INFOGEST static *in vitro* simulation of gastrointestinal food digestion. Nat Protoc. (2019) 14:991–1014. doi: 10.1038/s41596-018-0119-1 30886367

[76] Minekus M Alminger M Alvito P Ballance S Bohn T Bourlieu C . A standardised static *in vitro* digestion method suitable for food – an international consensus. Food Funct. (2014) 5:1113–24. doi: 10.1039/c3fo60702j, 24803111

[77] Lafarga T Rodríguez-Roque MJ Bobo G Villaró S Aguiló-Aguayo I. Effect of ultrasound processing on the bioaccessibility of phenolic compounds and antioxidant capacity of selected vegetables. Food Sci Biotechnol. (2019) 28:1713–21. doi: 10.1007/s10068-019-00618-4, 31807344 PMC6859129

[78] Guo W Mehrparvar S Hou W Pan J Aghbashlo M Tabatabaei M . Unveiling the impact of high-pressure processing on anthocyanin-protein/polysaccharide interactions: a comprehensive review. Int J Biol Macromol. (2024) 270:132042. doi: 10.1016/j.ijbiomac.2024.132042, 38710248

[79] Xue H Liang B Ji L Li X Wang M Liao X . The structure-activity relationship of polysaccharides in fruits and vegetables and interaction between polysaccharides and anthocyanins/proteins: a review. Food Res Int. (2025) 211:116371. doi: 10.1016/j.foodres.2025.116371, 40356164

[80] Kırnapcı M Berkel Kaşıkçı M. Antosiyaninlerin Gıda Matrisindeki Diğer Bileşenlerle Etkileşimlerinin Antosiyaninlerin Sindirim ve Emilimlerinin Üzerine Etkileri. Uşak Üniversitesi Fen ve Doğa Bilimleri Dergisi. (2025) 9:144–55. doi: 10.47137/usufedbid.1730491

[81] Khouryieh H. Impact of high pressure processing on the safety and quality of food products: a review. Recent Adv Food Nutr Agricult. (2024) 16:31–40. doi: 10.2174/012772574X289005240215093457 38409706

[82] Ahmed MW Gul K Mumtaz S. Recent advances in cold atmospheric pressure plasma for *E. coli* decontamination in food: a review. Plasma. (2025) 8:18. doi: 10.3390/plasma8020018

[83] Fan K Wu J Chen L. Ultrasound and its combined application in the improvement of microbial and physicochemical quality of fruits and vegetables: a review. Ultrason Sonochem. (2021) 80:105838. doi: 10.1016/j.ultsonch.2021.105838, 34801817 PMC8605411

[84] Dai Y Zhao X Zuo J Zheng Y. Effect of 100% oxygen-modified atmosphere packaging on maintaining the quality of fresh-cut broccoli during refrigerated storage. Foods. (2023) 12:1524. doi: 10.3390/foods12071524, 37048346 PMC10094251

[85] Sarron E Gadonna-Widehem P Aussenac T. Ozone treatments for preserving fresh vegetables quality: a critical review. Foods. (2021) 10:605. doi: 10.3390/foods10030605, 33809297 PMC8000956

[86] Athanasiadis V Chatzimitakos T Kotsou K Kalompatsios D Bozinou E Lalas SI. Polyphenol extraction from food (by) products by pulsed electric field: a review. Int J Mol Sci. (2023) 24:15914. doi: 10.3390/ijms242115914, 37958898 PMC10650265

[87] Wan Z Wang Y Shi X Hu W Zhao Y Liu X . Rheological properties of dysphagia food purees prepared according to IDDSI framework. Int J Food Prop. (2025) 28, 1–15. doi: 10.1080/10942912.2025.2462091

[88] Llavata B Mello RE Quiles A Correa JLG Cárcel JA. Effect of freeze-thaw and PEF pretreatments on the kinetics and microstructure of convective and ultrasound-assisted drying of orange peel. npj Sci Food. (2024) 8:56. doi: 10.1038/s41538-024-00301-x, 39181898 PMC11344832

[89] Souza FEB Rodrigues S Fonteles TV. Non-thermal technologies in food fermentation: mechanisms, benefits, and industrial perspectives for sustainable development. Processes. (2025) 13:2988. doi: 10.3390/pr13092988

[90] Sasi A Zainy NKA John A Shaji A Anujeslet VV Mohan J . Hurdle technology prototype to reduce microbial load in clarified citrus juice. Int J Curr Microbiol Appl Sci. (2024) 13:50–8. doi: 10.20546/ijcmas.2024.1310.007

[91] Melios S Stramarkou M Grasso S. Innovations in food: a review on the consumer perception of non-thermal processing technologies. LWT. (2025) 223:117688. doi: 10.1016/j.lwt.2025.117688

[92] Matías C Pereira-Caro G José Sáiz-Abajo M Cid C Ludwig IA Paz De Peña M. High-pressure and thermal pasteurization applied to smoothies enhances (poly)phenol bioaccessibility along the gastrointestinal tract. J Agric Food Chem. (2025) 73:15561–78. doi: 10.1021/acs.jafc.4c09166, 40497562 PMC12203591

[93] Rashvand M Dehkharghanian N Nikzadfar M Javed T Luke LP O’Brien A . Machine learning-driven optimization for digital transformation in non-thermal food processing. Food Bioprocess Technol. (2025) 18:10283–316. doi: 10.1007/s11947-025-04078-z

[94] Shanker MA Rana SS. Prospects of cold plasma in enhancing food phenolics: analyzing nutritional potential and process optimization through RSM and AI techniques. Front Nutr. (2025) 11:1504958. doi: 10.3389/fnut.2024.1504958, 39882036 PMC11774703

[95] Agrawal K Goktas P Holtkemper M Beecks C Kumar N. AI-driven transformation in food manufacturing: a pathway to sustainable efficiency and quality assurance. Front Nutr. (2025) 12:1–15. doi: 10.3389/fnut.2025.1553942, 40181942 PMC11966451

[96] Dhal SB Kar D. Leveraging artificial intelligence and advanced food processing techniques for enhanced food safety, quality, and security: a comprehensive review. Discov Appl Sci. (2025) 7:75. doi: 10.1007/s42452-025-06472-w

[97] Mahato DK Kamle M Pandhi S Pandey S Gupta A Paul V . Foodomics: a sustainable approach for the specific nutrition and diets for human health. Food Chem X. (2024) 24:101872. doi: 10.1016/j.fochx.2024.101872, 39483356 PMC11525469

[98] Yang M Wang Q. Carbon footprint and cost analysis of non-thermal food processing technologies: a review with a case study on orange juice. Front Sustain Food Syst. (2025) 9:1585467. doi: 10.3389/fsufs.2025.1585467

[99] Numa IAN Sancho RAS Wolf KE da Silva Miran CTC Soares SD de Souza Lima A . Polyphenols, aging, and health: what can we expect from the food industry in the technology era? Front Med (Lausanne). (2025) 12:1671886. doi: 10.3389/fmed.2025.1671886 41282017 PMC12630120

[100] Beaver LM Jamieson PE Wong CP Hosseinikia M Stevens JF Ho E. Promotion of healthy aging through the Nexus of Gut microbiota and dietary phytochemicals. Adv Nutr. (2025) 16:100376. doi: 10.1016/j.advnut.2025.100376, 39832641 PMC11847308

[101] Miguel MG. Betalains in some species of the Amaranthaceae Family: a review. Antioxidants. (2018) 7:53. doi: 10.3390/antiox7040053, 29617324 PMC5946119

[102] Ishida M Hara M Fukino N Kakizaki T Morimitsu Y. Glucosinolate metabolism, functionality and breeding for the improvement of Brassicaceae vegetables. Breed Sci. (2014) 64:48–59. doi: 10.1270/jsbbs.64.48, 24987290 PMC4031110

[103] Rolnik A Olas B. The plants of the asteraceae family as agents in the protection of human health. Int J Mol Sci. (2021) 22:1–10. doi: 10.3390/ijms22063009, 33809449 PMC7999649

[104] Aćimović MG. "Nutraceutical potential of apiaceae". In: Bioactive Molecules in Food (2019). p. 1311–41. The Netherlands: Springer Science and Business Media B.V.

[105] Asadi M Rasouli F Amini T Hassanpouraghdam MB Souri S Skrovankova S . Improvement of photosynthetic pigment characteristics, mineral content, and antioxidant activity of lettuce (*Lactuca sativa* L.) by arbuscular mycorrhizal fungus and seaweed extract foliar application. Agron. (2022) 12:1943. doi: 10.3390/agronomy12081943

[106] Čeryová N Lidiková J Grygorieva O Brindza J Demianová A Jurčaga L . Nutritional composition, polyphenol content, and antioxidant activity of Swiss chard (*Beta vulgaris* L. subsp. cicla). Agrobiodivers Improving Nutr Health Life Qual. (2025) 9:128–35. doi: 10.15414/ainhlq.2025.0014

[107] Bennett SD Littrell KW Hill TA Mahovic M Behravesh CB. Cold plasma from flexible and conformable paper-based electrodes for fresh produce sanitation: evaluation of microbial inactivation and quality changes. Food Control. (2022) 137:108915. doi: 10.1016/j.foodcont.2022.108915, 25167220

[108] Ali N Popović V Koutchma T Warriner K Zhu Y. Effect of thermal, high hydrostatic pressure, and ultraviolet-C processing on the microbial inactivation, vitamins, chlorophyll, antioxidants, enzyme activity, and color of wheatgrass juice. J Food Process Eng. (2020) 43:e13036. doi: 10.1111/jfpe.13036

[109] Giannoglou M Stergiou P Dimitrakellis P Gogolides E Stoforos NG Katsaros G. Effect of cold atmospheric plasma processing on quality and shelf-life of ready-to-eat rocket leafy salad. Innov Food Sci Emerg Technol. (2020) 66:102502. doi: 10.1016/j.ifset.2020.102502

[110] Hinojosa-Luna A Cámara-Martos F Pérez-Rodríguez F Serrano S Rodríguez I. High-pressure processing of freshly cut vegetables: influence on organoleptic properties, microbial load and trace element bioaccessibility. J Sci Food Agric. (2026) 106:3625–37. doi: 10.1002/jsfa.70480, 41603058

[111] Sarkar T Salauddin M Roy S Chakraborty R Rebezov M Shariati M. A. . Underutilized green leafy vegetables: frontier in fortified food development and nutrition. In Critical Reviews in Food Science and Nutrition. (2023) 63:11679–11733. Taylor and Francis Ltd. doi: 10.1080/10408398.2022.2095555 35816152

